# *In vivo* anticoccidial activity and immune response of *Moringa oleifera* and *Vernonia amygdalina* leaves against *Eimeria magna* and *Eimeria media* in rabbits

**DOI:** 10.3389/fcimb.2023.1173138

**Published:** 2023-05-18

**Authors:** Basile Konmy, Pascal Abiodoun Olounladé, Tomabu Adjobimey, Christian Cocou Dansou, Akouavi Carine Chimène Adoho, Esaïe Tchetan, Georcelin Goué Alowanou, Erick Virgile Bertrand Azando, Adolphe Adjanohoun, Sanni-yo Doko Allou, Lamine Baba-Moussa

**Affiliations:** ^1^Zootechnical Research and Livestock System Unit, School of Management and Operation of Livestock Systems, National University of Agriculture, Porto-Novo, Benin; ^2^Laboratory of Biology and Molecular Typing in Microbiology, Faculty of Sciences and Techniques, University of Abomey-Calavi, Abomey-Calavi, Benin; ^3^Laboratory of Ecology, Health and Animal Production, Faculty of Agronomy, University of Parakou, Parakou, Benin; ^4^Laboratory of Ethnopharmacology and Animal Health, Faculty of Agronomic Sciences, University of Abomey-Calavi, Cotonou, Benin; ^5^Unité de Biochimie et de Biologie Moléculaire, Faculté des Sciences et Techniques, Université d’Abomey-Calavi, Abomey-Calavi, Benin

**Keywords:** anticoccidial activity, immune response, *Moringa oleifera*, *Vernonia amygdalina*, *Eimeria magna*, *Eimeria media*

## Abstract

**Introduction:**

The aims of this study were to determine the immune response and the anticoccidial activity induced by *Moringa oleifera* and *Vernonia amygdalina* leaves in rabbits infected with *Eimeria magna* and *Eimeria media*.

**Methods:**

Thirty-five-day-old rabbits, free from coccidia, were infested with 2.10^3^ oocysts of *Eimeria magna* and *Eimeria media*, then received the acetone extract of the leaves of *Moringa oleifera* and *Vernonia amygdalina* at different doses by oral gavage.

**Results and discussion:**

The inhibition of the excretion of oocysts was evaluated by the McMaster technique and the levels of cytokines (IL-4 and IL-12) and immunoglobulin IgG were assayed by the ELISA method. The *in vivo* efficacy on *E. magna* and *E. media* oocysts was 95.43% and 96.53% for *Moringa oleifera* and *Vernonia amygdalina* at 1000 mg/kg bw against 98% for the positive control. Interestingly the plant extracts increased the production of interleukin (IL) and immunoglobulins (Ig) compared to controls. Plasma IL-4 levels (pg/ml) in rabbits were 128.94 and 131.38; those of IL-12 (pg/ml) were 395.55 and 426.56, and then for those of IgG (μg/ml) were 14.70 and 13.94 respectively with the acetone extracts of *Moringa oleifera* and *Vernonia amygdalina* on D14 PT at 1000 mg/kg bw. This study indicates that *Moringa oleifera* and *Vernonia amygdalina* can be used as an alternative to synthetic anticoccidials. These plants could be used to increase the resistance of the immune system of rabbits to infestations of Eimeria species in rabbit farms.

## 1 Introduction

Coccidiosis due to *Eimeria spp* is the most common parasitosis in rabbit farms. Every year, coccidiosis causes large economic losses in the rabbit industry (Xie et al., 2021). Obligate intracellular parasites, *Eimeria spp* infect epithelial cells (Tao et al., 2017a). For instance, *Eimeria intestinalis* infection destroys intestinal epithelial cells and triggers an inflammatory response in the gut. In addition, it induces a T helper 1 (Th1) immune response and increases the levels of pro-inflammatory and anti-inflammatory cytokines such as tumor necrosis factor alpha (TNF-α), interferon gamma (IFN-γ), interleukin 12 (IL-12), IL-6, and IL-10 (Abdel-Haleem et al., 2017). The anarchic and abusive use of veterinary anticoccidials in farms has produced problems of multiresistance and ecotoxicity. In the context of One Health, the increasing development of drug-resistant Coccidia species that have become a public health problem (Devi et al., 2010; Danquah et al., 2012) and leads to reflection on the search for an alternative substitute that respects the environment. The use of medicinal plants endowed with antiparasitic and immunological properties would constitute a promising way of combating coccidiosis.

*Vernonia amygdalina*, is commonly known as bitter leaves, belongs to the family Asteraceae, and is a rapidly regenerating shrub (Shui et al., 2021). This plant is widely distributed in the tropical rainforest of sub-Saharan Africa, Southeast Asia, and southern coastal regions of China (Igile et al., 1994). Historically, *V. amygdalina* was planted as a widely used tropical horticultural crop as a medicinal herb, food, and vegetable in Asia and Africa. *Vernonia amygdalina* has multiple pharmacological properties including antiparasitic, antihelminthic, antifungal, antimalarial, antiviral, anticancer, antibacterial, antimutagenic and antidiabetic properties (Atangwho et al., 2013; Asante et al., 2016; Alara et al., 2020). It is a tropical horticultural plant widely used around the world as a medicinal herb, vegetable, and animal and human food (Shui et al., 2021).

Furthermore *M. oleifera* known as “the miracle tree” has leaves rich in bioactive compounds such as flavonoids, polyphenols (Abidin et al., 2022) associated with numerous pharmacological effects such as antibacterial, antioxidants, antifungal and anti-inflammatory effects (El-Kassas et al., 2020). *Moringa oleifera* is a prebiotic and improves animal health and growth performance (Vongsak et al., 2013).

These two plants while their efficacy demonstrated in resolving health issues, their antiparasitic effects and their impact on immune system of rabbits remain to be investigated. The objective of this study is to determine the anticoccidial activity and the immune response induced by *Moringa oleifera* and *Vernonia amygdalina* in rabbits infected with *Eimeria magna* and *Eimeria media*.

## 2 Method

### 2.1 Isolation and preparation of *Eimeria magna* and *Eimeria media*

2 ml of a solution containing 3,10^4^ sporulated oocysts from naturally infested rabbits was orally inoculated into 5 healthy rabbits of 60-days-olf and weighing 1.5-1.8 kg. After 3 days of infestation, qualitative coproscopy was performed daily to confirm infestation. 9 days after infestation, the inoculated rabbits were sacrificed and the contents of the jejunum and ileum were recovered. *E. magna* and *E. media* oocysts were identified and isolated from these contents using the method described in previous studies Coudert et al. (1995). Morphometric characters of sporulated oocysts Oocyst size (filtration), duration of pre-patent period (to separate species with shorter endogenous development), sporulation time (to separate species with shorter sporulation time), differential tropism for intestinal segments were used for identification according previous study (Coudert et al., 1995) and counting was done using the McMaster technique. Then, oocysts were mixed with a 2.5% potassium dichromate (K_2_Cr_2_O_7_) and incubated at 25°C. The mixture was aerated and examined daily for sporulation (Tao et al., 2017b; Eladl et al., 2020). After complete sporulation, the oocysts were washed by centrifugation several times until the supernatant became clear; then, the supernatant was removed and the pellet stored at 4° C until further use. The culture of the oocysts of *Eimeria magna* and *Eimeria media* previously obtained was done in healthy rabbits during 5 generations (Eladl et al., 2020) before the infestation of the subjects used for the in this study.

### 2.2 Vegetable material

*Moringa oleifera* and *Vernonia amygdalina* leaves were collected at the University of Abomey-calavi. These sheets have been identified at the National Herbarium of the University of Abomey-Calavi under the voucher numbers N°AA66/1645/HNB; and YH523/HNB respectively. These leaves were washed with water and dried in the laboratory at 18°C until they became crisp. The dried leaves were then pulverised with a grinder and stored in airtight jars for later use.

### 2.3 Preparation of the acetone extract

50 g of plant leaf powder was suspended in an acetone/water mixture (70:30, v/v, 500 ml) for 2 hours. The mixture was then filtered through Watman No. 1 brand filter paper. The resulting filtrate was evaporated under vacuum using a Rotavapor (BUCHI RII) at 47° C. and then dried in the oven. The yield of the dry extracts obtained from each plant was calculated. The extracts obtained were stored at 4°C and used for phytochemical and biological analyses.

### 2.4 Animals and management

Ninety-six New Zealand White rabbits, 35 days old, of both sexes (48 males and 48 females), with 625 ± 85 g average body weight were used. Rabbits were housed in individual galvanized cages 80 cm wide × 50 cm long × 40 cm high. These animals were acclimatized for 14 days and qualitative fecal coproscopies were performed and the technique of concentration by flotation (He and Venant, 2004; El-Shahawi et al., 2012) was used on each subject prior to their use in this study. Coproscopies were performed daily for 14 successive days to confirm that the animals were free of any coccidia oocysts. The general conditions of hygiene, equipment and animal handling complied with international bioethical standards (Lozoya et al., 1990). the experiments were conducted at the animal facilities of the faculty of agronomic sciences of the university of Abomey-Calavi.

### 2.5 Experimental design

Forty-eight rabbits of both sexes were subdivided into 8 groups. Each group is composed of 6 rabbits. These 48 rabbits were used for one species of Eimeria parasite. All groups were infested with 2.10^3^ sporulated oocysts of *Eimeria magna* according to (Licois, 2010). The groups M_A_; M_B_ and M_C_ were treated with acetone extract of *Moringa oleifera* at 1000 mg; 500 mg and 250 mg/kg body weight, respectively. The groups V_A_; V_B_ and V_C_ were treated with acetone extract of *Vernonia amygdalina* at 1000 mg; 500 mg and 250 mg/kg body weight, respectively. The positive control group T_P_ was treated with amprocox 5 mg/kg bw and the Negative Control group T_N_ received distilled water. The experiment was faithfully repeated with the remaining 48 rabbits each infested with 2.10^3^ sporulated oocysts of *Eimeria media* as described above. Rabbits were treated after 7 days of infestation with *Eimeria magna* and after 5 days of infestation with *Eimeria media* according to the prepatent period.

### 2.6 Parasitological examination of faeces

Faeces samples were collected using a device placed under the cages, then placed in polythene bags and transferred to the laboratory at 4°C. These samples were collected at 0, 2-, 4-, 7-, and 14-days post infestation. The McMaster technique was used for the determination of the number of oocysts per gram (OPG) of faeces. Thus, 4g of fresh rabbish faeces were crushed in a mortar and mixed with. 56 ml of a saturated NaCL solution of specific gravity 1.2; then, the mixture was filtered. through a tea strainer. After homogeneisation, the chambers of the McMaster slide were filled and allowed to stand 5 mn before reading on microscope at 10x or 4x magnification.

#### 2.6.1 Oocyst reduction rate

The percentage of oocyst reduction was determined according to the formula:


$$Reduction\;rate\;of\;oocysts=\frac{Initial\;average\;OPG\;-Final\;average\;OPG\;}{Initial\;average\;OPG}x100$$

#### 2.6.2 Assessment of growth performance

The body weight (bw) of the rabbits was recorded weekly. The Feed Conversion Ratio (FCR) was calculated by dividing the weight of feed consumed by the average weight gain. The rabbits were macroscopically examined daily. The mortality rate was recorded.

### 2.7 Assessement serum immunoglobulin G

Sera from all rabbits were collected prior to infestation and weekly after infestation. All serum samples were stored at -20°C. IgG were assessed by Sandwich ELISA technique, using ELISA Cusabio kits (Cusabio, Wuhan, China). The intensity of the colorimetric reaction was measured using a MR-96A Microplate Reader.

### 2.8 Measurement of serum cytokine levels

Serum levels of rabbit interleukin (IL) IL-4 and IL-12 were assessed by Sandwich ELISA technique, using ELISA Cusabio kits (Cusabio, Wuhan, China) in accordance with the manufacturer’s instructions. It is Sandwich ELISA. The intensity of the colorimetric reaction was measured using a MR-96A Microplate Reader.

### 2.9 Hematological and biochemical examination

At the end of the experiment, blood samples were collected in Ethylene Diamine Tetra-acetic Acid (EDTA) tubes for hematological analyses and in dried tubes for biochemical analyses. Blood samples were used to determine leukocytes, lymphocytes, and erythrocyte counts, as well as liver enzyme profiles, including ALT, AST, GGT, and PAL activities, and total protein levels.

### 2.10 Statistical analysis

All data were analyzed using SPSS version 26.0 software (IBM SPSS, Chicago, IL, USA) and GraphPad Prism version 9.0.2 software (GraphPad Software, USA). The Shapiro-Wilk test was performed and since the distribution of variables was normal, data were compared using two-way analysis of variance (ANOVA) and followed by the Tukey *post-hoc* multiple range test. All data are expressed as mean ± Standard Error Mean (SEM). p-value<0.05 was considered as significant.

## 3 Results

### 3.1 Clinical signs

The prepatent period in the rabbits of the groups having been infested with *Eimeria magna* was 7 days post-infestation (PI), whereas it was 5 days PI for the subjects having been infested with *Eimeria media*. In the two untreated control groups of the two experiments, the clinical signs observed were depression, diarrhea, emaciation, and abdominal distension throughout the experiment. In addition, the groups treated with plant extracts; M_A-C_ and V_A-C_ presented slight emaciation and abdominal distension and diarrhea. These clinical signs appeared in the PI period and gradually resolved to disappear 5 days Post-Treatment (PT).

### 3.2 Mortality rate

No death was recorded in the treated groups and in the positive control group throughout the duration of the experiment. In the negative control group, no deaths were recorded during the first 7 days PI. However, during the experiment, the mortality rate in the negative control group was 37.5% for those who received *Eimeria media* oocysts and 62.5% for those who received *Eimeria magna* oocysts.

### 3.3 *In vivo* anticoccidial activity

**Figure 1:**
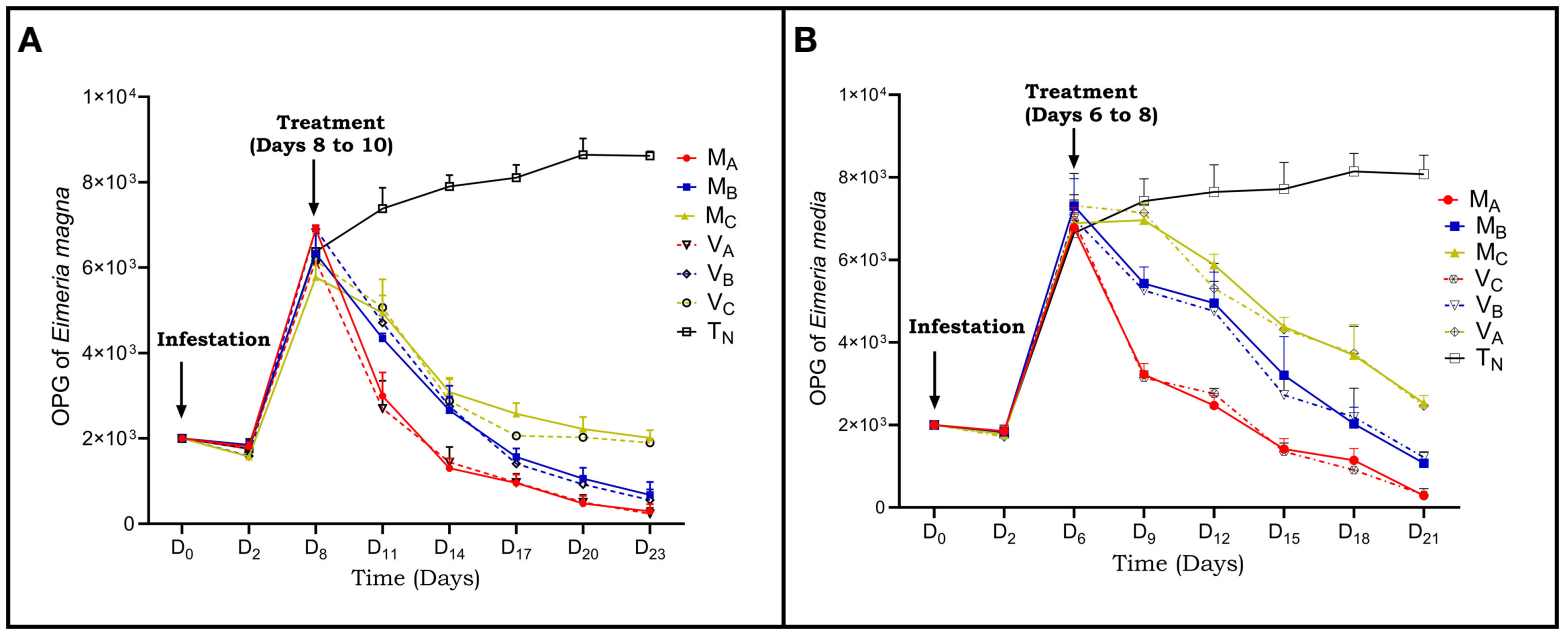
Evolution of oocysts per gram of faeces of rabbits infected and treated with acetone extracts of *Moringa oleifera* and *Vernonia amygdalina*. **(A)** infestation with *Eimeria magna* oocysts; **(B)** infestation with *Eimeria media* oocysts. M_A_, Acetone extract of *Moringa oleifera* at 1000 mg/kg body weight, M_B_, Acetone extract of *Moringa oleifera* at 500 mg/kg body weight; M_C_, Acetone extract of *Moringa oleifera* at 250 mg/kg body weight; V_A_, *Vernonia amygdalina* acetone extract at 1000 mg/kg body weight; V_B_, Acetone extract of *Vernonia amygdalina* at 500 mg /kg body weight; V_C_, *Vernonia amygdalina* acetone extract at 250 mg/kg body weight, T_N_, Negative control. Values are means ± SEM (n = 6).

The number of oocysts in the negative control group T_N_ continuously increased throughout the duration of the experiment. On the other hand, the final faecal oocyst count of rabbits infected with *Eimeria magna* or *Eimeria media* and treated with *Moringa oleifera* and *Vernonia amygdalina* leaves extracts in different groups showed a significant (p<0.001) dose-dependent reduction in the number of oocysts in treated groups after 14 days (**Figures 1A, B**).

On D11 and D9, which represent 3 days PT, respectively, with *Eimeria magna* and *Eimeria media*, statistical differences (P<0.05) in the mean number of OPG between the rabbits in the groups receiving 250 mg/kg; 500 mg/kg and 1000 mg/kg bw of acetone extract of each plant for the two species of parasites.

On D23 and D21 (14 days PT with *Eimeria magna* and *Eimeria media* respectively), a decrease (P<0.001) in the number of oocysts was obtained in the groups receiving 1000 mg/kg of extract of *Moringa oleifera* and *Vernonia amygdalina* compared to the group that received 250 mg/kg.

#### 3.3.1 Effectiveness of plant extracts

**Figure 2:**
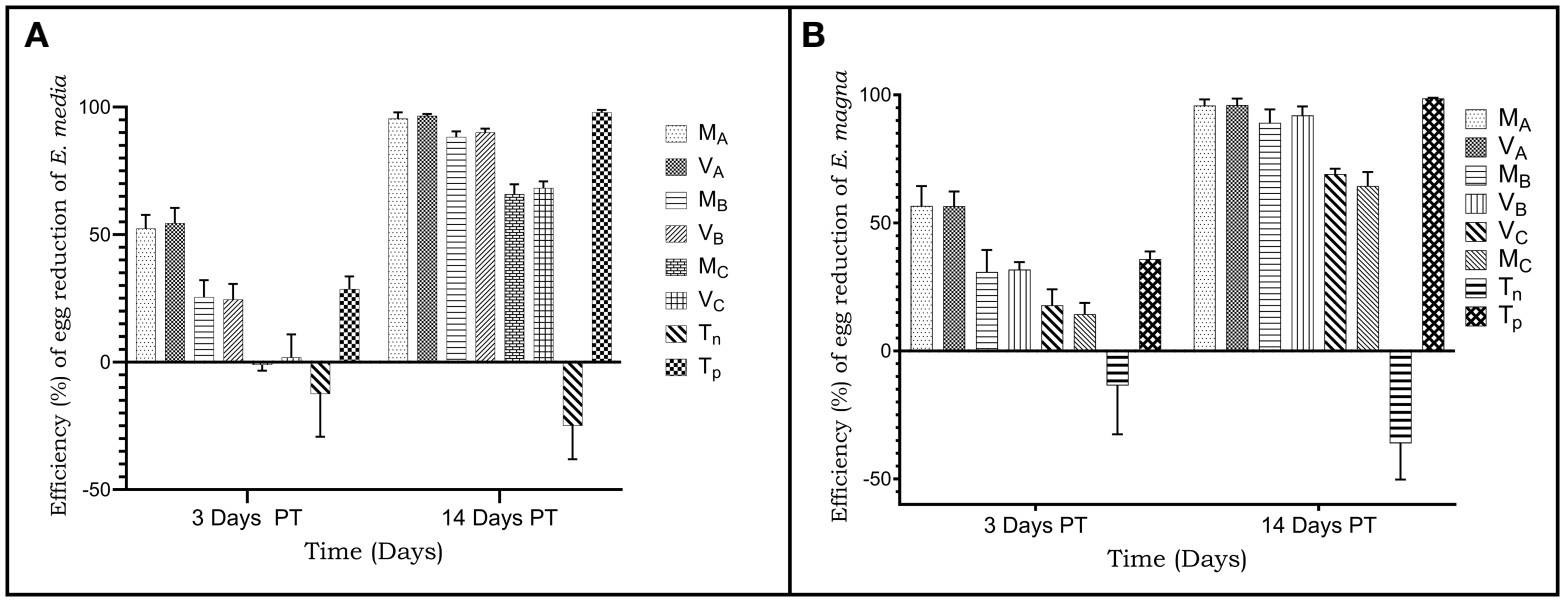
Reduction rate (%) of oocysts per gram of faeces from rabbits infected and treated with acetone leaves extracts of *Moringa oleifera* and *Vernonia amygalina.* **(A)** infestation with *Eimeria media* oocysts; **(B)** infestation with *Eimeria magna* oocysts. M_A_, Acentone extract of *Moringa oleifera* at 1000 mg/kg body weight; M_B_, Acetone extract of *Moringa oleifera* at 500 mg/kg body weight; M_C_, Acetone extract of *Moringa oleifera* at 250 mg/kg body weight; V_A_, *Vernonia amygdalina* acetone extract 1000 mg/kg body weight; V_B_, Acentone extract of *Vernonia amygdalina* at 500 mg/kg body weight; V_C_, *Verninia amygdalina* acetone extract at 250 mg/kg body weight; T_N_, Negative control; T_P_, Positive control treated with Amprocox at 5 mg/kg bw. Values are means ± SEM (n = 6).

In rabbits infested with *Eimeria media* oocysts, the highest reduction rate was 97.87% in the positive control group. The oocyst reduction rate was 95.43%; 88.26% and 65.83%, respectively, with 1000 mg dose; 500 mg and 250 mg/kg of *Moringa oleifera* extract. The oocyst reduction rate was 96.53%; 90.05% and 68.26%, respectively, with 1000 mg doses; 500 mg and 250 mg/kg *Vernonia amygdalina* extract. The reduction rates were dose dependent regardless of the plant extract (**Figure 2A**).

The rate of reduction in the number of oocysts of animals infected with *Eimeria magna* oocysts was 100% in the positive control group. Oocyst reduction rates were 95% and 96% (1000 mg/kg); 89.07% and 91.94% (500 mg/kg), and then 64.39% and 69.01% (250 mg/kg) respectively with the extracts of *Moringa oleifera* and *Vernonia amygdalina* (**Figure 2B**).

#### 3.3.2 Weight gain of infested rabbits.

The first week of the experiment (D0-6 for the subjects infested by *E. media* and D0-8 for the subjects infested by *E. magna*) corresponded to the week of infestation of the rabbits with the strains of *Eimeria media* and *Eimeria magna*. During this week, the subjects of the different infested groups experienced a weight loss which was not significantly different (p>0.05) between the groups (**Table 1**). Weight loss ranged between -53 g and 71 g for subjects infested with *Eimeria media* and then from -93 g to 108 g for subjects infested with *Eimeria magna*. During the two weeks following the treatment with the plant extracts, a significant improvement (p<0.01) in the weight gain of the test subjects was observed, while in the negative control subjects no improvement was observed. The improvement in weight gain between the treated groups was not significant during this period. The best weight increases were obtained in subjects treated with 1000 mg of plant extracts (**Table 1**).

**Table 1:** Weight gain of rabbits infested with *Eimeria media* and *Eimeria magna* and then treated with acetone extracts of *Moringa oleifera* and *Vernonia amygdalina*.

	Infestation with *Eimeria media*			Infestation with *Eimeria magna*		
	D0-6	D7-13	D14-21	D0-7	D7-15	D15-23
M_A_	-53 ± 7.49	73.33 ± 3.11b	167.17 ± 6.02d	-96 ± 14.64	117 ± 16.50a	172 ± 15.62a
M_B_	-64.67 ± 11.26	64.67 ± 4.94cb	119 ± 4.82a	-100.33 ± 12.25	109 ± 6.43a	158.67 ± 21.42a
M_C_	-55.50 ± 10.83	72.50 ± 10.25b	106.17 ± 2.77ab	-93.67 ± 6.17	106.33 ± 8.29a	130 ± 16.26a
V_A_	-60 ± 3.82	88.17 ± 3.38b	156.50 ± 7.76d	-90.67 ± 21.73	112 ± 11.72a	178.67 ± 23.02a
V_B_	-64.50 ± 5.35	98.67 ± 5.63cb	126.17 ± 14.47ab	-95 ± 14.98	107 ± 15.18a	154.33 ± 5.55a
V_C_	-66.50 ± 8.89	95.17 ± 11.85cb	98.17 ± 11.40ab	-108 ± 6.25	104 ± 12.58a	115.33 ± 24.59a
T_P_	-70 ± 9.01	108.50 ± 14.95d	123.50 ± 21.23b	-101 ± 13.65	101.33 ± 15.76a	144 ± 10.44a
T_N_	-71.50 ± 7.40	-87.83 ± 3.35a	-60 ± 11.07c	-98 ± 23.76	-108.33 ± 11.89b	-110 ± 28.29b
pvalue	0.731425	3.8147E-10	8.0343E-9	0.995630	1.1954E-8	2.9484E-7

M_A_, Moringa oleifera at 1000 mg/kg bw; M_B_, Moringa oleifera at 500 mg/kg bw; M_C_, Moringa oleifera at 250 mg/kg bw of acetone extract. V_A_, Vernonia amygdalina at 1000 mg/kg bw; V_B_, Vernonia amygdalina at 500 mg/kg bw; V_A_, Vernonia amygdalina at 250 mg/kg bw TP, Positive control; TN, Negative control. For the same column and the same period, the values with the same superscript letter are not significantly different at p ≥ 0.05 (Duncan’s test). The means in the same row affected by the different letters “a b and c” are significantly different (P<0.05).

### 3.4 Determination of antibody and cytokine levels

**Figure 3:**
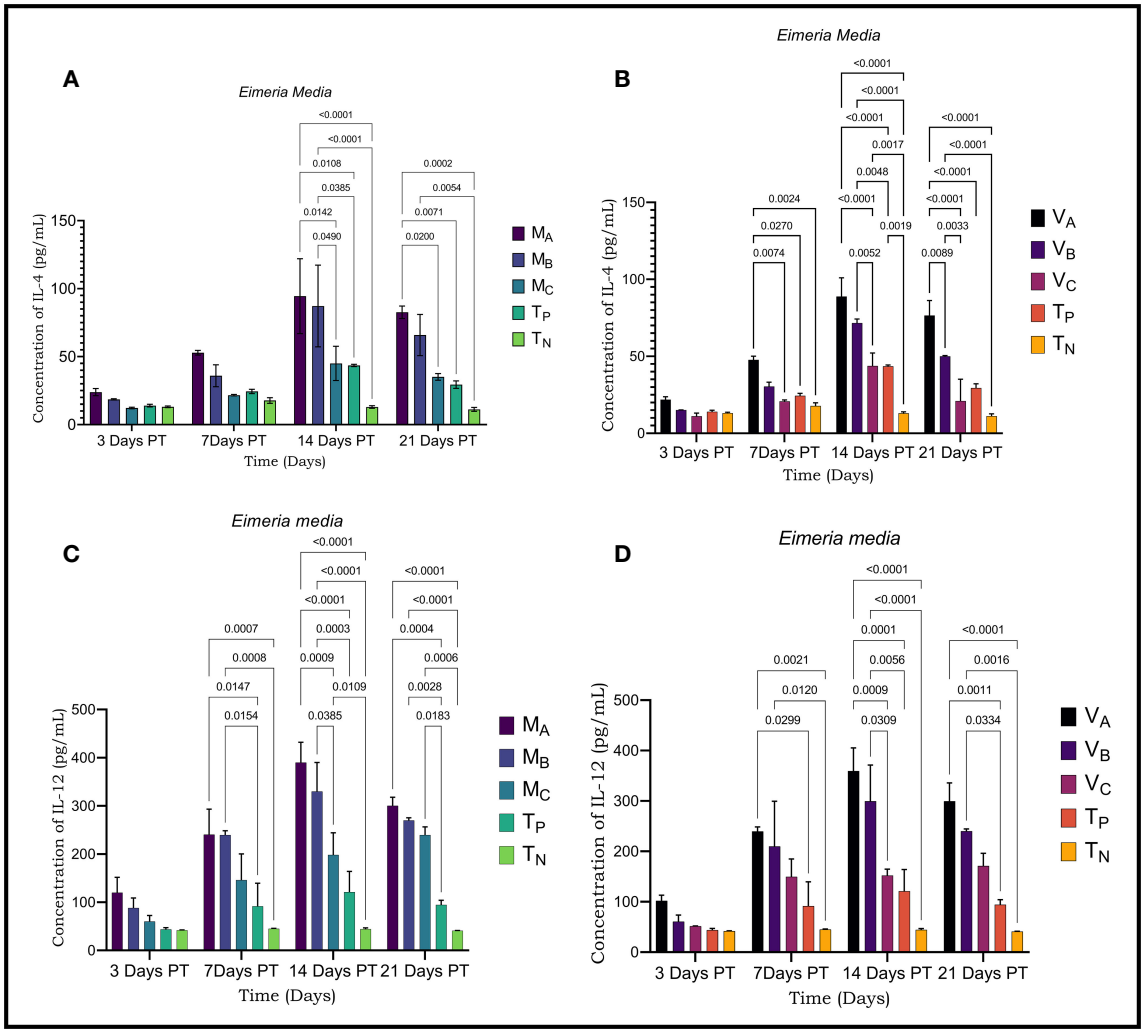
Result of cytokine and immunoglobulin levels assessed by ELISA in rabbits infected with *Eimeria media.* **(A)** Levels of IL-4 secreted by rabbits infested with *E*. *media* and treated with *Moringa oleifera* leaves extract **(B)**; Rates of IL-4 secreted by rabbits infected with *E*. *media* and treated with *Vernonia amygdalina* leaves extract; **(C)**; Levels of IL-12 secreted by rabbits infested with *E*. *media* and treated with *Moringa oleifera* leaves extract **(D)**; Levels of IL-12 secreted by rabbits infected with *E*. *media* and treated with *Vernonia amygdalina* leaves extract. M_A_, Acetone extract of *Moringa oleifera* at 1000 mg/kg body weight; M_B_, Acetone extract of *Moringa oleifera* at 500 mg/kg body weight; M_C_, Acetone extract of *Moringa oleifera* at 250 mg/kg body weight; V_A_, *Vernonia amygdalina* acetone extract at 1000 mg/kg body weight; V_B_, Acetone extract of *Vernonia amygdalina* at 500 mg/kg body weight; V_C_, *Vernonia amygdalina* acetone extract at 250 mg /kg body weight; T_N_, Negative control; T_P_, Positive control treated with Amprocox at 5 mg/kg bw. Values are means ± SEM (n =6). These indicators were detected using kits obtained from CUSABIO (CUSABIO, Wuhan, Chinan). p-value <0.05 was considered as significant (Tukey's test).

**Figure 4:**
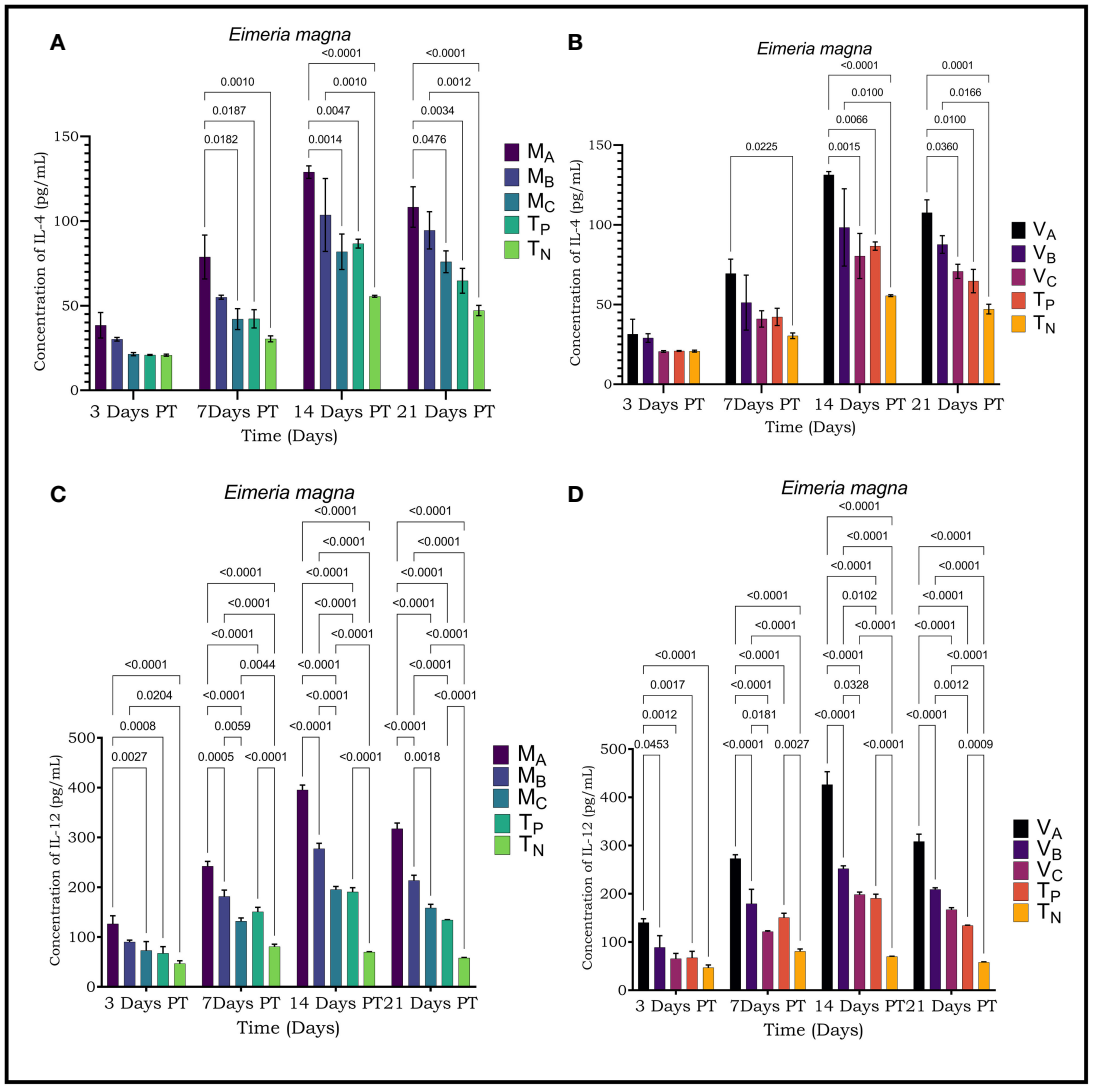
Levels of cytokines IL-4 and IL-12 were evaluated by ELISA in rabbits infected with *Eimeria magna*. **(A)** Levels of IL4 secreted by rabbits infested with *E*. *magna* and treated with *Moringa oleifera* leaves extract **(B)**; Levels of IL-4 secreted by rabbits infested with *E*. *magna* and treated with *Vernonia amygdalina* leaves extract; **(C)**; Levels of IL-12 secreted by rabbits infested with *E*. *magna* and treated with *Moringa oleifera* leaves extract **(D)**; Levels of IL-12 secreted by rabbits infested with *E*. *magna* and treated with *Vernonia amygdalina* acetone leaves extract. M_A_, Acetone extract of *Moringa oleifera* at 1000 mg/kg body weight; M_B_, Acetone extract of *Moringa oleifera* at 500 mg/kg body weight; M_C_, Acetone extract of *Moringa oleifera* at 250 mg/kg body weight; V_A_, *Vernonia amygdalina* acetone extract at 1000 mg/kg body weight; V_B_, Acetone extract of *Vernonia amygdalina* at 500 mg/kg body weight; V_C_, *Vernonia amygdalina* acetone extract at 250 mg/kg body weight; T_N_, Negative control; T_P_, Positive control treated with Amprocox at 5 mg/kg bw. Values are means ± SEM (n =6). These indicators were detected using kits obtained from CUSABIO (CUSABIO, Wuhan, Chinan). p-value <0.05 was considered as significant (Tukey's test).

**Figure 5:**
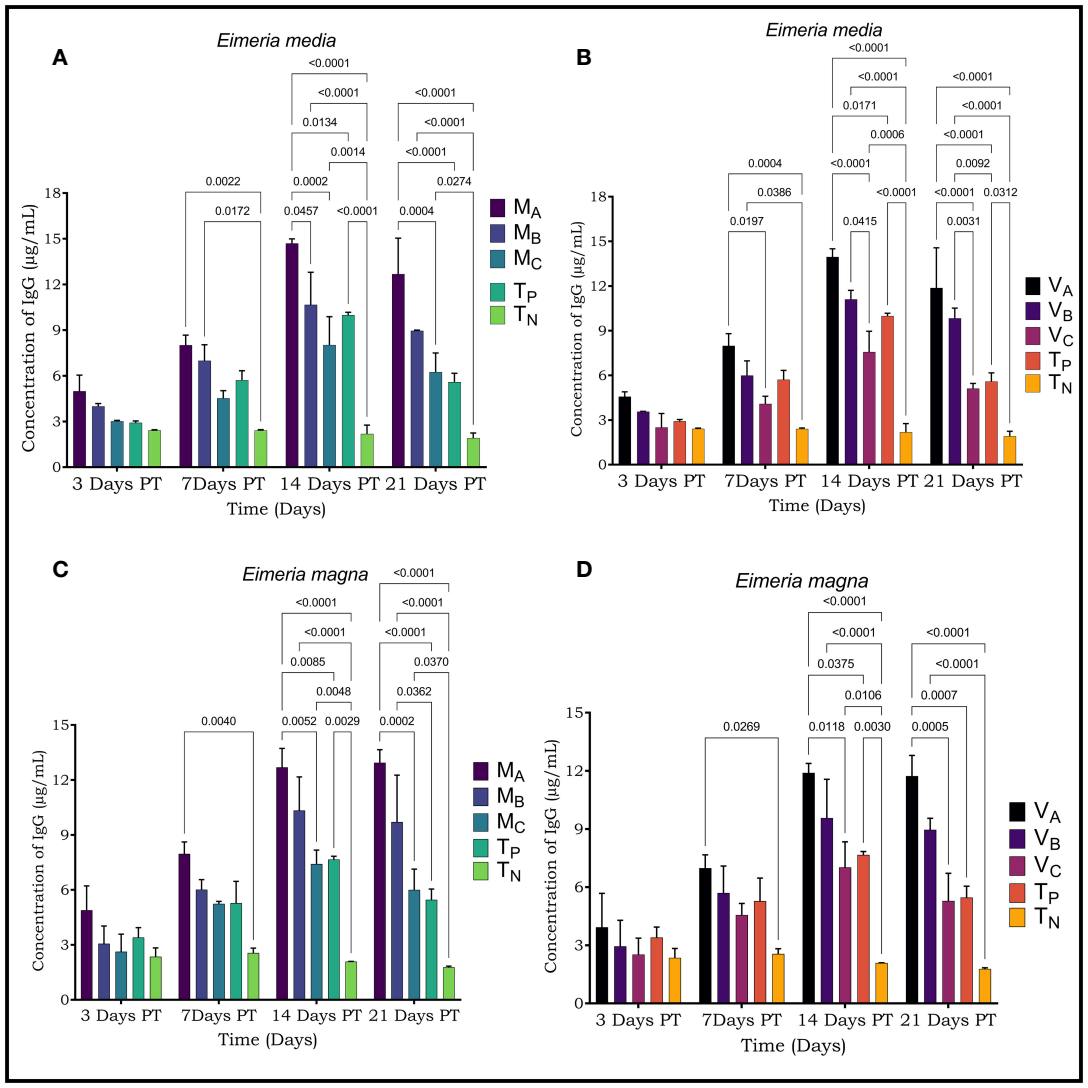
Immunoglobulin G (IgG) levels were assessed by ELISA in rabbits infected with *Eimeria media* and *Elmeria magna*. **(A)** Level of IgG secreted by rabbits infested with *E*. *media* and treated with *Moringa oleifera* leaf extract **(B)**; Levels of IgG secreted by rabbits infected with *E*. *media* and treated with *Vernonia amygdalina* leaf extract; **(C)**; Levels of IgG secreted by rabbits infested with *E*. *magna* and treated with *Moringa oleifera* leaf extract **(D)**; Levels of IgG secreted by rabbits infested with *E*. *magna* and treated with *Vernonia amygdalina* leaf extract. M_A_, Acetone extract of *Moringa oleifera* at 1000 mg/kg body weight; M_B_, Acetone extract of *Moringa oleifera* at 500 mg/kg body weight M_C_, Acetone extract of *Moringa oleifera* at 250 mg/kg body weight; V_A_, *Vemonia amygdalina* acetone extract at 1000 mg/kg body weight; V_B_, Acetone extract of *Vemonia amygdalina* at 500 mg/kg body weight; V_C_, *Vernonia amygdalina* acetone extract at 250 mg/kg body weight: T_N_: Negative control; T_P_, Positive control treated with Amprocox at 5 mg/kg bw. Values are means ± SEM (n=6). These indicators were detected using kits obtained from CUSABIO (CUSABIO, Wuhan, China). value <0.05 was considered as significant (Tukey's test).

The concentrations of cytokines (IL-4 and IL-12) and immunoglobulins (IgG) of sera from rabbits treated with plants were determined from the regression equation of the calibration curves of the standards of each parameter (**Figures 3**–**5**).

Significant differences were detected in plasma levels of IL-4 and IL-12 in infected and treated rabbits compared to negative controls. Plasma levels of IL-4 and IL-12 varied during the experiment. IL-4 and IL-12 values increased gradually over time and this increase was dose-dependent. The plasma concentrations of these cytokines showed a very significant change on D14 and D21 PT (p<0.001) where they reached their highest values in *Eimeria media* as with *Eimeria magna*. However, at D21 PT, the plasma levels of IL-4 and IL-12 decreased compared to D14 PT. The highest cytokine level was observed at the concentration of 1000mg/kg. For rabbits infested with *Eimeria media*, the plasma level of IL-4 was 94.47 ± 27.52 pg/ml (D14 PT); 82.62± 4, 57 (D21 PT) with *Moringa oleifera* extracts and then 88.85± 12.15 pg/ml (D14 PT) and 76.53± 9.73 (D21 PT) with *Vernonia amygdalina* extract at a dose of 1000 mg/kg body weight (**Figures 3A, B**). Plasma levels of IL-12 were 389.70 ± 42.17 pg/mL (D14 PT) and 300.20 ± 17.60 pg/ml (D21 PT) with *Moringa oleifera* extract and then 359.60 ± 45.71 pg/ml (D14 PT) and 299.45 ± 36.33 pg/mL (D21 PT) with *Vernonia amygdalina* extract (**Figures 3C, D**).

Plasma levels of IL-4 in rabbits infected with *Eimeria magna* were 128.94 ± 3.68 pg/ml on D14 PT; 108.29 ± 11.97 pg/ml on D21 PT 131.38 ± 2.01 pg/ml on D14 PT; 107.59 ± 8.06 pg/ml on D21 PT respectively with the extracts of *Moringa oleifera* and *Vernonia amygdalina* (**Figures 4A, B**). Regarding IL-12, the plasma levels were 395.55 ± 9.54 pg/ml on D14 PT; 317.61 ± 11.40 pg/ml on D21 PT and then 426.56 ± 26.68 pg/ml on D14 PT; 308.66 ± 14.83 pg/ml at D28 PT respectively in the extracts of *Moringa oleifera* and *Vernonia amygdalina* (**Figures 4C, D**).

A humoral response was induced in rabbits infected with *Eimeria media* and in rabbits infected with *Eimeria magna*. IgG immunoglobulin values were higher in *Eimeria media* and *Eimeria magna* at D14 and D21 posttreatment (PT). The IgG value in infected and treated rabbits was higher than in infected and untreated rabbits. The highest IgG concentrations were found at D14 PT. Moreover, this value was significantly different between the treated rabbits and the controls (p<0.001) on the one hand and between the rabbits treated with different concentrations of plant leaves extracts (p<0.05).

### 3.5 Hematological and biochemical profile of animals

On rabbits infested with *E. media*, the number of white blood cells (WBC) experienced a significant change (p<0.03) compared to rabbits of negative control group. In opposite, the number of red blood cells, haemoglobin level, haematocrit level, and blood platelets showed a non-significant variation (p>0.05) (**Table 2**). About rabbits infected with *E. magna*, White blood cells (p<0.0001), haemoglobin level (p<0.001), and haematocrit level (p<0.001) showed a significant variation compared to subjects in negative control group. However, other parameters such as red blood cells and blood platelets (p>0.05) did not show any significant variation (**Table 3**).

**Table 2:** Hematological parameters of rabbits infested with *Eimeria media* and then treated with extracts of *Moringa oleifera* and *Vernonia amygdalina*.

	M_A_	V_A_	M_B_	V_B_	M_C_	V_C_	T_P_	T_N_	SEM	pvalue
Red Blood Cells (10^6^/mm^3^)	4.66	4.84	4.69	4.86	4.83	4.86	4.88	4.83	0.11	0.916
Haemoglobin (g/dl)	11.03	10.32	10.63	10.33	10.65	10.23	10.83	9.90	0.28	0.311
Packed Volume Cell (%)	33.80	31.63	32.53	31.27	32	31.07	32.47	30.07	0.85	0.229
Blood Platelets (10^3^/mm^3^)	138.33	116.33	113.83	119.67	141.33	104.33	153.67	124.33	15.48	0.478
White Blood Cell (10^3^/L)	9.60a	9a	6.97ab	7ab	6.60ab	6.41ab	7.20ab	4.17b	0.82	0.03
Neutrophils	39.67	35.67	36.33	33.67	37.33	32.67	36	31.67	1.91	0.271
Eosinophils	2.33	1.67	2.17	2	2.33	2	2.33	2	0.33	0.935
Basophils	0	0	0	0	0	0	0	0	0	
Lymphocytes	53.67	58	57	60.50	57.33	60.67	58	62	1.93	0.26
monocytes	4.33	4.67	4.50	3.50	3	4.67	3.33	4.33	0.49	0.216

M_A_, Acetone extract of Moringa oleifera at 1000 mg/kg of body weight; M_B_, Acetone extract of Moringa oleifera at 500 mg/kg body weight; M_C_, Acetone extract of Moringa oleifera at 250 mg/kg body weight; V_A_, Vernonia amygdalina acetone extract at 1000 mg/kg body weight; V_B_, Vernonia amygdalina acetone extract at 500 mg/kg body weight; V_C_, Vernonia amygdalina acetone extract at 250 mg/kg body weight; T_N_, Negative control; T_P_, Positive control treated with Amprocox at 5 mg/kg bw. The means in the same row affected by the different letters “a and b” are significantly different (P<0.05).

**Table 3:** Hematological parameters of rabbits infested with *Eimeria magna* and then treated with extracts of *Moringa oleifera* and *Vernonia amygdalina*.

	M_A_	V_A_	M_B_	V_B_	M_C_	V_C_	T_P_	T_N_	SEM	pvalue
Red Blood Cells (10^6^/mm^3^)	5.41	4.94	5.05	4.33	4.87	4.51	4.72	4.32	0.21	0.112
Haemoglobin (g/dl)	13.90a	14.13a	11.93ab	11.90ab	10.70ab	11.13ab	13.13a	8.57b	0.60	0.001
Packed Volume Cell (%)	41.70a	42.40a	35.80ab	35.70ab	32.10ab	33.40ab	39.40a	25.70b	1.80	0.001
Blood Platelets (10^3^/mm^3^)	155	159	154	168.33	169	174.67	159.67	176	11.00	0.855
White Blood Cell (10^3^/L)	10.09c	9.68c	7.90b	7.62b	7.56b	7.34b	8.19b	6.03a	0.37	0.001
Neutrophils	39.33	39	39.33	37.67	40	36.33	36.33	31	3.16	0.7
Eosinophils	2	3	2.33	2	2.33	3	2.33	2.67	0.16	0.041
Basophils	0	0	0	0	0	0	0	0	0	.
Lymphocytes	54.33	54.67	55	56.67	53.67	56.67	57.67	62.33	2.99	0.71
monocytes	4.33	3.33	3.33	3.67	4	4	3.67	4	0.39	0.715

M_A_, Acetone extract of Moringa oleifera at 1000 mg/kg of body weight; M_B_, Acetone extract of Moringa oleifera at 500 mg/kg body weight; M_C_, Acetone extract of Moringa oleifera at 250 mg/kg body weight; V_A_, Vernonia amygdalina acetone extract at 1000 mg/kg body weight; V_B_, Vernonia amygdalina acetone extract at 500 mg/kg body weight; V_C_, Vernonia amygdalina acetone extract at 250 mg/kg body weight; T_N_, Negative control; T_P_, Positive control treated with Amprocox at 5 mg/kg bw. The means in the same row affected by the different letters “a b and c” are significantly different (P<0.05).

The aspartate aminotransferase (AST); alanine aminotransferase (ALT); alkaline phosphatase (ALP); urea and creatinine levels did not vary significantly (p>0.05) in all treated and untreated rabbits. However, the total protein level increased significantly (p< 0.001) in all infected and treated rabbits compared to controls (**Tables 4**, **5**).

**Table 4:** Biochemical parameters of rabbits infested with *Eimeria media* and then treated with extracts of *Moringa oleifera* and *Vernonia amygdalina*.

	M_A_	V_A_	M_B_	V_B_	M_C_	V_C_	T_P_	T_N_	SEM	pvalue
Urea (g/L)	0.34	0.32	0.4	0.39	0.37	0.46	0.48	0.53	0.0475	0.051
Creatinine (g/L)	9.23	10.52	12.03	12.18	12.68	14.02	13.42	16.27	0.24625	0.826
AST (IU)	37.33	33	31	36	40.67	32.33	45.67	34.67	5.58125	0.676
ALT (IU)	80.06	66	62.5	55.83	76	49	68.67	51	9.0725	0.275
ALP (IU)	195	218.67	216.5	186.17	191	209.33	153.67	191.67	21.80375	0.601
Total protein (g/L)	90.67b	89b	86.25b	84.50b	80.50b	82.33b	84b	61a	3.10125	0.001

M_A_, Acetone extract of Moringa oleifera at 1000 mg/kg body weight; M_B_, Acetone extract of Moringa oleifera at 500 mg/kg body weight; M_C_, Acetone extract of Moringa oleifera at 250 mg/kg body weight; V_A_, Vernonia amygdalina acetone extract at 1000 mg/kg body weight; V_B_, Vernonia amygdalina acetone extract at 500 mg/kg body weight; V_C_, Vernonia amygdalina acetone extract at 250 mg/kg body weight; T_N_, Negative control; T_P_, Positive control treated with Amprocox at 5 mg/kg bw. The means in the same row affected by the different letters “a and b” are significantly different (P<0.05).

**Table 5:** Biochemical parameters of rabbits infested with *Eimeria magna* and then treated with *Moringa oleifera* and *Vernonia amygdalina* extracts.

	M_A_	V_A_	M_B_	V_B_	M_C_	V_C_	T_P_	T_N_	SEM	pvalue
Urea (g/L)	0.28	0.28	0.32	0.29	0.32	0.39	0.42	0.51	0.0525	0.552
Creatinine (g/L)	11.08	9.51	12.34	12, 02	13.22	12.67	15.35	18, 45	0.17875	0.243
AST (IU)	38.42	67.05	38.72	58.36	102.24	88.22	23.42	29.99	21.22625	0.347
ALT (IU)	86.2	88.81	85.75	75.36	72.64	78.38	93.15	88.68	5.84375	0.331
ALP (IU)	167.33	167	169.33	116.67	117.67	123.67	166	126.33	24.1625	0.731
Total protein (g/L)	89c	94.33c	74b	75.33b	67.67b	68.67b	76.67b	53a	2.4725	0.001

M_A_, Acetone extract of Moringa oleifera at 1000 mg/kg body weight; M_B_, Acetone extract of Moringa oleifera at 500 mg/kg body weight; M_C_, Acetone extract of Moringa oleifera at 250 mg/kg body weight; V_A_, Vernonia amygdalina acetone extract at 1000 mg/kg body weight; V_B_, Vernonia amygdalina acetone extract at 500 mg/kg body weight; V_C_, Vernonia amygdalina acetone extract at 250 mg/kg bw; T_N_, Negative control; T_P_, Positive control treated with Amprocox at 5 mg/kg bw. The means in the same row affected by the different letters “a and b” are significantly different (P<0.05).

## 4 Discussion

This study aimed to evaluate the anticoccidial potential and determine the immune response induced by *Moringa oleifera* and *Vernonia amygdalina* leaves for the treatment of rabbits infected by *Eimeria media* and *Eimeria magna*.

After the infestation of group of rabbits with one of the two species of Eimeria and their treatment with the leaves extract of one of the plants at different concentrations, the survival rates, diarrhea, body weight, feed conversion rate and oocyst excretion were studied. In the different treated groups, all parameters were improved compared to the negative control group. Plant extracts and amprocox had similar effects in rabbits after infestation with *E. media* or *E. magna*.

This study demonstrates that treating rabbits with herbal powder extract significantly reduced diarrhea in infected rabbits. Indeed, reducing diarrhea can help strengthen the infested animal to better fight off secondary bacterial invasion and inflammatory reactions (Muthamilselvan et al., 2016). Thus, the antidiarrheal activity of extracts of *Moringa oleifera* and *Vernonia amygdalina* can be attributed to therapeutic effects such as reduction of intestinal motility and direct anti-coccidial activity. It may be due to the fact that these plants contain a large amount of quercetin (a flavonoid) which prevents the release of acetylcholine in the digestive tract, a hypothesis supported by (Giannenas et al., 2020). The number of oocysts in faeces is a crucial indicator of the spread of coccidiosis in farms due to the transmission of Eimeria oocysts by the faecal-oral route. According to reports from previous studies (Cervantes-Valencia et al., 2015; Cedric et al., 2017; Aboelhadid et al., 2019; Qaid et al., 2021), the number of faecal oocysts in rabbits infested with coccidia is effectively reduced in response to the medicinal plants used.

In the present study, higher doses of the acetone extract of *Moringa oleifera* and *Vernonia amygdalina* administered showed better anticoccidial activity in rabbits infected with *Eimeria media* and *Eimeria magna*. This anticoccidial activity resulted in improved weight gain and a significant (p<0.01) reduction in OPG. This reduction in OPG was dose-dependent. The reduction in the number of oocysts of both Eimeria species probably indicates that the extracts of *Moringa oleifera* and *Vernonia amygdalina* prevent the development of the parasites in the host before the relatively inert oocysts are formed and eventually released. The reduction rates at the level of groups treated with plant extracts, indicating that the highest prophylactic efficacy among all these groups was recorded in the groups that received the highest dose of plant extracts. These results are similar to those of Cedric et al. (2017) who studied the *in vivo* anticoccidial and antioxidant activity of methanolic extracts of Psidium guajava on *Eimeria intestinalis* oocysts. They are also in good agreement with the results of Cervantes-Valencia et al. (2015) who studied the influence of Curcuma longa as a natural anticoccidial in adult rabbits. Qaid et al. (2021) claim that *Rumex nervosus* powder, at a dose of 5 g, has moderate anti-coccidial effects and could therefore be used to treat avian coccidiosis in farms.

The growth performance of subjects in the infested and treated groups was affected. Those infested and untreated were more affected. These results are in good agreement with the results of previous work (Cedric et al., 2017) which showed that rabbits infested with *Eimeria intestinalis* had a significant reduction in body weight and food consumption compared to uninfected rabbits. This decrease in performance can lead to poor nutrient absorption and reduced immune response and, consequently, intestinal tissue damage (Adhikari et al., 2020).

The results of this study show that the growth performance of subjects in all groups was negatively affected by infestation with *Eimeria magna* or *Eimeria media* before the start of treatment. However, it was found that during treatment body weight, growth rate, and feed conversion was significantly improved (p< 0.01) by treatment with plant extracts and amprocox compared to witness. This result agrees with the results reported by (Cedric et al., 2017; Qaid et al., 2021) respectively in rabbits and chicken. In the present study, it was noticed that the evolution of performance was dose-dependent as described by the results of (Cervantes-Valencia et al., 2015), showing that performance increased with increasing dose.

The increase in inhibition of oocyst excretion and growth performance parameters were proportional to the dose. These results contrast with those of (Qaid et al., 2021)which showed that the performance parameters of *Rumex nervosus* leaves powder in chickens were disproportionate to dose.

Cellular immune responses play a dominant role in immunity against coccidiosis (Chapman, 2014). Eimeria infection in rabbits damages the jejunal structure, and increases intestinal inflammation, and disrupts gut microbiota and metabolic homoeostasis (Yuan et al., 2022). Altered faecal microbiota and metabolites are potential biomarkers of coccidiosis infestation (Yuan et al., 2022). In this study, the cellular immune responses induced by *Eimeria magna* and *Eimeria media* in the presence of *Moringa oleifera* and *Vernonia amygdalina* leaves extracts were evaluated. Two interleukins, IL-4 and IL-12, were measured in the plasma of the subjects tested. IL-4 and IL-10 are secreted by Th2-like cells and regulate humoral immunity, effectively aiding in B cell activation (Inagaki-Ohara et al., 2006). Elevated levels of Th1-type (IL-12) and Th2-type (IL-4) cytokines were quantified. These subject-induced immune responses resulted in effective protection against Eimeria. These results are consistent with other reported results. Several cytokines have been shown to be involved in immune responses to Eimeria infection (Chapman, 2014). Th1-type cytokines such as IL-12 are responsible for cellular immunity and dominate during Eimeria infection. In the present study, the production of IL-12 by the cells is much higher than that of IL-4. This explains why the cellular reaction is more solicited than the humoral one in an Eimeria infection. The immune reaction to *Eimeria spp* would stimulate Th1-type cytokines and cells much more than Th2 cells. This result agrees with the results of the work of (Pakandl et al., 2008). The results of the IL-4 level of this study are superior to those of the work of (Li et al., 2015) in a study performed on rabbits and rats. This result could be explained by the fact that the plant extracts used to treat the infested rabbits better stimulate the production of IL-4. The level of IgG immunoglobulins determined in this study in infected and treated rabbits is higher than the level of IgG determined in the work of (Pakandl et al., 2008) in a study performed on rabbits infested with *Eimeria flavescens* and *Eimeria intestinalis*. This difference could be explained by the fact that *Moringa oleifera* and *Vernonia amygdalina* have the capacity to boost the immune system of rabbits during an infestation with *Eimeria* spp. Also in the previous study Pakandl et al. (2008) had not treated the infested animals. The trend towards an increase in Th1 cytokines indicates that the immune response of TCD8 cells could be the most solicited. IL-4, a typical Th2-type cytokine, is responsible for stimulating humoral immunity (Inagaki-Ohara et al., 2006).

## 5 Conclusion

This study shows that the acetonic extract of *Moringa oleifera* and *Vernonia amygdalina* significantly inhibited the excretion of *Eimeria magna* and *Eimeria media* oocysts in rabbits. The effect of the extracts was observed from day 3 after treatment until day 21. The 500 mg/kg and 1000 mg/kg concentrations of the two plants had the best inhibition efficacy. The detection of cytokine levels reveal that the plant extracts better stimulate the immune system *via* the cellular response. *Moringa oleifera* and *Vernonia amygdalina* leaves could be an alternative to coccidiosis in organic rabbit farming.

## Data availability statement

The raw data supporting the conclusions of this article will be made available by the authors, without undue reservation.

## Ethics statement

The animal study was reviewed and approved by the study was carried out in strict compliance with the recommendations of the guide of the Research Ethics Committee of the National University of Agriculture (UNA), Porto Novo, Republic of Benin and in line with detailed protocols of Animal Care and Use in Research, Education and Testing: N° 062- 2016/P-Ethic Committee/SA. Written informed consent was obtained from the owners for the participation of their animals in this study.

## Author contributions

BK, CCD, EVBA and PAO designed the study. BK, ACCA and ET reviewed the literature; wrote the manuscript BK, CCD, SDA performed experiments and analyzed data. BK, ET, TA and PAO performed statistical analysis. BK revised the manuscript. AA, TA, GGA, PAO, SDA, EVBA and LBM, Supervised AA, PAO, SDA, LBM; All authors contributed to the article and approved the submitted version.
